# The Cytotoxic Effectiveness of Thiourea-Reduced Graphene Oxide on Human Lung Cancer Cells and Fungi

**DOI:** 10.3390/nano13010149

**Published:** 2022-12-28

**Authors:** Babu Vimalanathan, J. Judith Vijaya, B. Carmel Jeeva Mary, Ruby Nirmala Mary, Mohamed Km, Ramasamy Jayavel, Rasha A. Abumousa, Mohamed Bououdina

**Affiliations:** 1 Crystal Growth Centre, Anna University, Chennai 600025, Tamil Nadu, India; 2 Catalysis and Nanomaterials Research Laboratory, Department of Chemistry, Loyola College, Chennai 600034, Tamil Nadu, India; 3 Department of Mathematics and Sciences, College of Humanities and Sciences, Prince Sultan University, Riyadh 11586, Saudi Arabia

**Keywords:** reduced graphene oxide, lung cancer cells, cell viability, DNA fragmentation, *Aspergillus flavus*, antifungal activity

## Abstract

This study demonstrated the effective reduction of graphene oxide (GO) by employing thiourea as a reducing and stabilizing agent. Two fungi (*Aspergillus flavus* and *Aspergillus fumigatus*) were used for anti-fungal assay. Cell viability, cell cycle analysis, DNA fragmentation, and cell morphology were assessed to determine the toxicity of thiourea-reduced graphene oxide (T-rGO) on human lung cancer cells. The results revealed that GO and T-rGO were hazardous to cells in a dose-dependent trend. The viability of both *A. fumigatus* and *A. flavus* was affected by GO and T-rGO. The reactive oxygen species produced by T-rGO caused the death of *A. flavus* and *A. fumigatus* cells. This study highlighted the effectiveness of T-rGO as an antifungal agent. In addition, T-rGO was found to be more harmful to cancer cells than GO. Thus, T-rGO manifested great potential in biological and biomedical applications.

## 1. Introduction

A planar sheet of carbon atoms with sp^2^ bonds that are closely together in a honeycomb crystal structure, known as graphene nanosheet, has received a great attention from both fundamental research in condensed matter and quantum field theory and potential technological applications [1]. Three bonds and one out-of-plane bond, which can bind with nearby atoms, are present in each carbon atom [2]. In combination with graphene’s electron distribution, this atomic structure gives the material exceptional mechanical qualities, great chemical stability, huge surface area, and high thermal and electrical conductivity.

Additionally, graphene oxide, reduced graphene oxide (rGO), single- as well as multilayered graphene, each of which has distinct adjustable features, can be fabricated from graphene sheets through chemical and physical modifications. Graphene has been extensively adopted for application in several areas since its experimental recognition in 2004 [3], including printable inks [4], electronics, energy, water, and biomedicine [5,6,7].

The biological uses of graphene oxide (GO), which exhibits functional groups including oxygen species, include cancer treatment, drug transport, biosensing, and tissue engineering [8,9,10]. The presence of oxygen functional groups makes substances more soluble in water and makes it possible to covalently functionalize substances with a variety of medicines, polymers, and fluorescent markers through diverse chemical pathways [11,12].

Graphene and its derivatives are extensively used in different industries, including electronics [13], energy storage and conversion [14], optics [15], and most recently, bioscience and biotechnology [16]. Due to their large surface area and outstanding thermal and electrical conductivity, they were employed in the creation of bio- and immune-sensors [17], DNA sensing [18], biocomposites [19], gene delivery [20], pathogen sensing at the single-cell level [21], tissue engineering [22], and antifungal properties [23].

In order to photolyze viruses, the GO-aptamer complex has been utilized as a catalyst [24], and graphene and GO have been recently used for virus detection. For rapid screening and suppression of the hepatitis C virus gene throughout liver cells [25], as well as for large-scale screening of inhibitors of the severe acute respiratory syndrome corona virus (SARS-CoV) helicase and the hepatitis C virus (HCV) NS3 helicase [26], the GO-based multiplexed helicase assay was developed.

The advantage of reduced graphene with thiourea is its cost-effectiveness. The functional improvements are excellent, including structural properties, mechanical strength, dispersibility, and reactivity.

The objective of this research work consisted of developing a straightforward, affordable, and scalable method for functionalizing and reducing GO using thiourea. Further, GO and thiourea-reduced GO (T-rGO) were tested against human lung cancer cell line (A549) by examining DNA fragmentation, cell cycle arrest, and apoptosis. In addition, the toxicity of GO and T-rGO was also assessed against two plant pathogenic fungi, *Aspergillus flavus* and *A. fumigates*. The T-rGO could be used as an effective antifungal and anticancer agent in nanotherapeutic strategies.

## 2. Materials and Methods

The following materials were used directly out of the bottle: graphite powder, analytical grade thiourea, NaOH, KMnO_4_, anhydrous ethanol, 98% H_2_SO_4_, 36% HCl, and 30% H_2_O_2_ aqueous solution. All aqueous solutions were created using deionized water (DW). All further chemicals, unless otherwise noted, were obtained from (Sigma Aldrich, Mumbai, India).

Dimethyl sulfoxide (DMSO), fetal bovine serum (FBS), and DMEM were purchased from Hi-Media Laboratories. DMSO, trypsin, and methylthiazolyldiphenyl-tetrazolium bromide (MTT) were bought from Cistron Lab (Sisco Research Lab Chemicals, Mumbai, India). The remainder of the chemicals and reagents were from (Sigma-Aldrich Mumbai, India).

### 2.1. Synthesis of GO

The modified Hummers’ approach successfully synthesized graphite oxide [27] using graphite powder as raw material with KMnO_4_, NaNO_3_, and 98% H_2_SO_4_ as oxidants.

### 2.2. Synthesis of T-rGO

An amount of 250 mL of DW was ultrasonically mixed with 500 mg of GO for 1.5 h. In a typical reduction experiment, 500 mg of thiourea was then added to the dispersion while it was being stirred, and it was then refluxed at 94 °C for 24 h. After going through the reduction procedure, the GO dispersion’s brown tint turned to black.

### 2.3. Characterization

The crystalline structure of GO and T-rGO was examined using powder X-ray diffraction (XRD) on Rigaku Miniflex II-Ce equipped with Cu-Kα radiation (λ = 0.15406 nm), as previously reported [28,29]. Employing Agiltron Peak Seeker+ro 532, Raman spectra were captured at room temperature with an excitation wavelength of 532 nm. Morphological findings were performed by Tescan vega 3 (Zeck Republic) scanning electron microscopy (SEM). Gold was applied to the GO and T-rGO powders after they had been mounted to an Al sample holder for imaging. With the use of the KBr technique, the Jasco 6600FT-IR spectrometer was able to identify the types of bonds and functional groups. Using a nitrogen atmosphere and a growth temperature of 10 °C/min up to 1000 °C, an EXSTAR SII TG/DTA 6300 was used to analyze the sample’s thermal stability.

### 2.4. Antifungal Assay

T-rGO nanosheets were mixed with 15 mL of PDA agar at 50 °C and added to previously sterilized petri dishes (95 mm in diameter). A young (5-day-old) PDA fungal culture had its agar discs (5 mm in diameter) coated in fungal growth aseptically removed after solidification (NOT CLEAR). In the middle of PDA agar plates containing T-rGO (25–500 µg/mL), the discs were aseptically positioned. All petri plates underwent a 7-day incubation at 25 °C. Five replicates were used in the experiments. The typical fungal colony diameters were then measured. The following formula was used to compute the antifungal impact, expressed as percentage of mycelia growth inhibition (*MGI*) [30]:

(1) $$MGI\left(\%\right)=\frac{d_{c}-d_{t}}{d_{t}}\times 100$$

where the average diameter of fungal colony in control and after treatment in mm is denoted by *d_c_*, and *d_t_*, respectively.

### 2.5. Cell Line and Culture

Human lung cancer cell line (A549) was offered by NCCS Pune. The cells were kept alive in DMEM media supplemented with penicillin (100 U/mL), 10% FBS, and streptomycin (100 U/mL) at 37 °C in a humidified atmosphere with 5% CO_2_ [31].

### 2.6. Culturing of Cells and Their Treatment with GO and T-rGO

The procedure for cell culture was carried out as previously described in the literature [31]. Human lung cancer cells were grown in an incubator at 37 °C with 5% CO_2_ in DMEM with 10% FBS, 2 mM glutamine, and 100 U/mL penicillin–streptomycin. The cells were passaged via the sub confluency after the culture medium was renewed three times weekly. An amount of 0.25% trypsin–ethylenediamine tetra acetic acid was used to collect the cells at about 90–95% confluency and kept in 96-well plates or 6-well plates and a 75 cm^2^ flask, as needed for the experiment. After 24 h, the medium was changed to a new medium containing GO and T-rGO at concentrations that ranged from 1000 to 7.8 µg/mL; the negative controls were the cells that had not been exposed to GO and T-rGO. After 24 h of incubation, the treated cells were checked for their viability, DNA fragmentation, stage in the cell cycle, and morphology.

### 2.7. WST-8 Cell Viability Test

Cell viability was assessed using the WST-8 test, as previously explained [32,33]. A quantity of 1 × 10^4^ cells of A549 was plated in 100 µL of DMEM medium (10% FBS) in a 96-well plate. The cells were then given two washes in 100 µL of serum-free DMEM media before being cultured in this medium with varying concentrations of GO or T-rGO suspensions. Following an exposure time of 24 h, the cells were washed twice with serum-free DMEM medium before being analyzed. After that, the WST-8 solution was added to each well that had been pre-filled with 100 µL of serum-free DMEM medium. Following an incubation period of one hour, 80 µL of the mixed solution was moved to a different 96-well plate. This was performed so that any leftover GO or T-rGO wouldn’t influence the absorbance measured at 450 nm using a microplate reader. Cell-free control assays determined whether GO and T-rGO directly responded to WST-8. A 96-well plate was typically filled with 100 L of GO or T-rGO suspensions of different concentrations, and 10 µL of WST-8 reagent solution was added to each well. This mixture was then incubated for 1 h at 37 °C with 5% CO_2_. After incubation, they were centrifuged, and 50 µL of each supernatant was then added to a fresh 96-well plate. The optical density was observed at 450 nm.

### 2.8. Cell Cycle Arrest Examination

Human lung cancer cells were plated at a density of 2.0 × 10^5^ cells/mL on a six-well plate. The cells were treated with GO and T-rGO at IC_50_ concentrations (62.5 µg/mL) in serum-free media and incubated for an additional 24 h at 37 °C with 5% CO_2_. The IC_50_ obtained from the cytotoxicity test for each treatment was utilized in the cell cycle experiment. Analyses of the cell cycle phases were performed in accordance with Anita et al. [34]. Prior to being collected, the cells were trypsinized and centrifuged at 1500 rpm for 5 min. Propidiumiodide (10 µg/mL) was added to 0.2 mL of cell suspension. After 30 min of incubation at 37 °C, the cells were checked with a flow cytometer at 488 nm for both excitation and emission to find out what stage of the cell cycle they were in. The presented findings are typical of at least three distinct tests performed in triplicate.

### 2.9. DNA Fragmentation

In order to achieve convergence, 2.0 × 10^5^ human lung cancer cells were plated in 6 well-plates and cultivated in a CO_2_ incubator. After achieving confluence, the cells were exposed to varying concentrations of GO and T-rGO (62.5 µg/mL is the IC_50_ concentration). An amount of 1.5 mL of the cell suspension from the TPVG-collected cells was then placed in an Eppendorff tube. Following that, the cells were centrifuged for 10 min at 20,000 rpm and 4 °C. After that, 0.5 mL of TTE solution was aggressively vortexed with the pellet. This method made nuclear structure breakdown owing to Mg^++^ chelation by EDTA in the TTE solution after cell lysis and the release of fragmented chromatin from nuclei easier, since Triton X-100 was included in the TTE solution. After carefully discarding the supernatant, 500 µL of TTE solution was added to the particle. Then, 500 micro liters of ice-cold NaCl were added and violently vortexed. DNA histones were removed with the addition of salt. Then, 700 µL of ice-cold isopropanol was provided, aggressively refluxed, and allowed to precipitate at 20 °C for a whole night. By centrifuging for 10 min at 20,000 rpm/4 °C and washing with 500–700 µL of ice-cold 70% ethanol, the DNA pellet was recovered. At 4 °C, the DNA pellet was dispersed in 20–50 µL of TE buffer. This DNA was electrophoresed using standard TE buffer. The DNA was mixed with loading buffer (10×) and bromophenol blue dye to reach a final concentration of (1×). This made it simpler to feed samples into the wells and track their movement over the gel. When the dye was approximately 3 cm from the end of the gel, electrophoresis was stopped. A UV trans-illuminator was utilized to see the gel [35,36].

### 2.10. Cell Morphology

After seeding 1 × 10^5^ cells/well of human lung cancer cells into 6-well plates, they were maintained in the incubator for 24 h. In different GO or T-rGO concentrations (1000, 62.5, and 7.8 µg/mL that is IC_50_ concentration), the cells were grown. Meanwhile, the control cells were grown without GO and T-rGO. Cells were exposed for 24 h, and their morphology was analyzed using an optical microscope [37].

## 3. Results and Discussion

### 3.1. XRD Analysis

**Figure 1:**
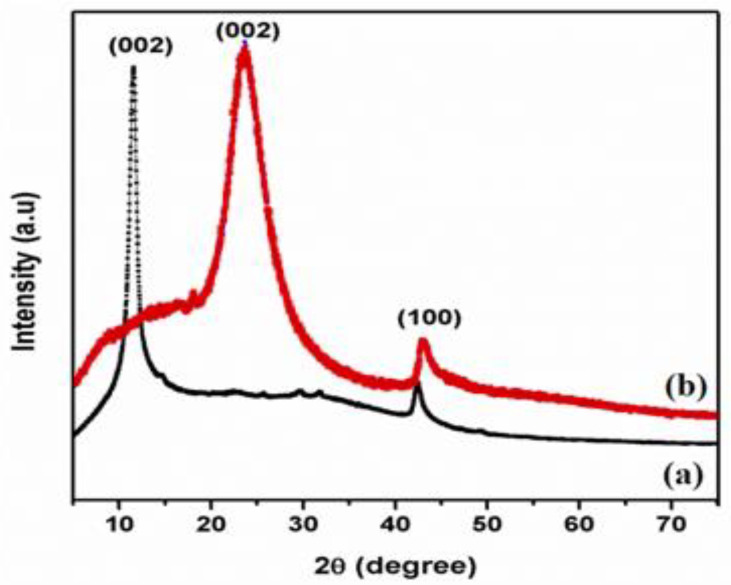
X-ray diffraction patterns of (a) GO and (b) T-rGO.

Figure 1 displays XRD pattern of GO and T-rGO powders. Exfoliated GO manifests a lower angle of diffraction than pristine graphite, i.e., 10.5° and 26°, respectively. The peak at 2θ = 10.5° is caused by the deposition of water molecules between the sheets of the graphite and the creation of functional groups containing oxygen, which caused the increase in d-spacing [38].

Figure 1 shows the XRD pattern of T-rGO. It reveals a broad peak between 2θ = 24° and 29° and a peak centered at 2θ = 25°, which means that the separation between the layers is 0.35 nm, which is different from pure graphite. When graphene oxide is reduced with thiourea, the broad peak previously observed at 2θ = 25° vanishes, demonstrating a substantial decrease in GO and the production of graphene with fewer layers [39,40].

### 3.2. SEM Analysis

**Figure 2:**
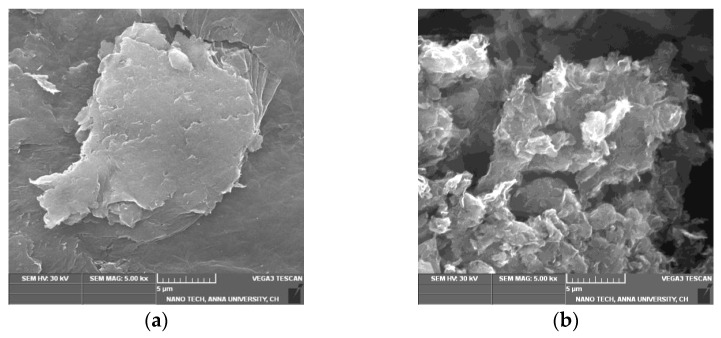
FESEM images of (**a**) GO and (**b**) T-rGO.

The SEM image of GO (Figure 2a) indicates that GO is composed of stacked and aggregated, thin, and crumbled sheets that are closely linked with one another in a disordered trend. The sheets have little folds at the edges and are smooth overall with minute wrinkles. After reduction, the SEM images of T-rGO (Figure 2b) show the presence of translucent plates and rigid silk sheet. The observed morphologies of both GO and T-rGO corroborate well with the literature [41,42].

### 3.3. FTIR Analysis

**Figure 3:**
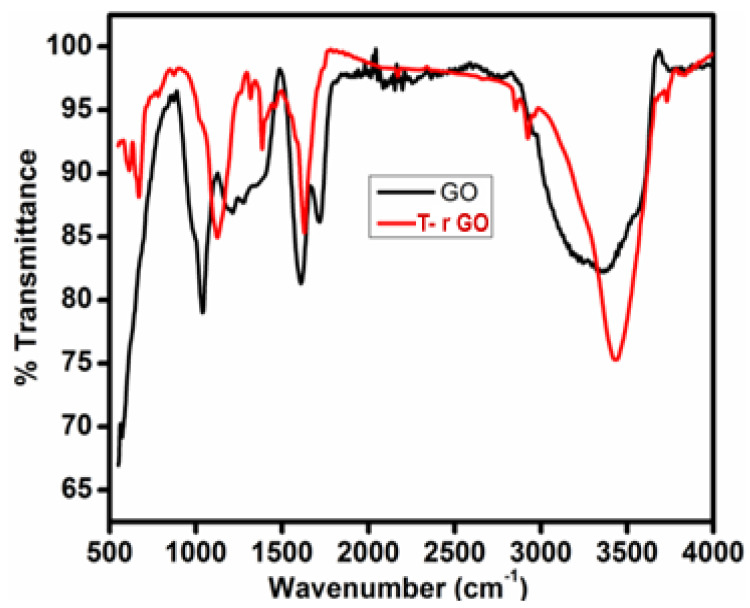
FTIR spectral analysis of (black line) GO and (red line) T-rGO.

The FTIR spectra of GO and T-rGO are shown in Figure 3. O-H group vibration and deformation, C=O stretching, C-O (epoxy) stretching, and C-O-C stretching correspond to the bands detected at 3365, 1729, 1230, and 1035 cm^−1^. The aforementioned bands indicate that GO contains a variety of oxygen-containing compounds. The FTIR spectrum of T-rGO differs significantly from that of GO. The emergence of the OH stretching band at 3424 cm^−1^ indicates that the amount of GO has been drastically reduced throughout the deoxygenating process (Figure 3). Overall, FTIR analysis shows that the oxygen-containing compounds have been completely eliminated during the reduction process of GO. This is consistent with the findings of the literature [43,44].

### 3.4. Thermal Analysis

**Figure 4:**
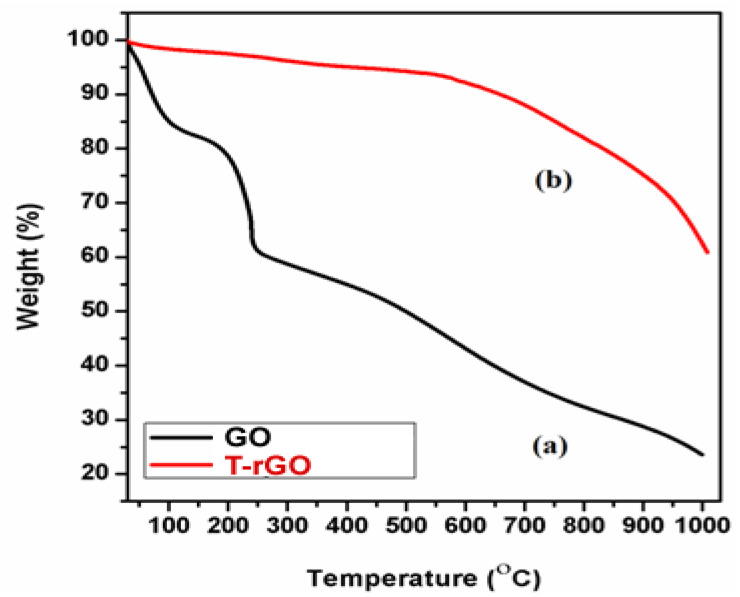
Thermogravimetric analysis of (a) GO and (b) T-rGO.

The thermal stability of the synthesized graphene derivatives GO and T-rGO was investigated using TGA analysis, as shown in Figure 4. The initial stage (150 and 200 °C) represents the removal of oxygen-containing functional groups, such as H_2_O and O_2,_ with a mass loss of 15% [45]. At temperatures exceeding 300 °C, the carbon skeleton’s bulk pyrolysis causes an additional 25% mass loss. T-rGO demonstrates much greater thermal stability than pure GO at temperatures above 600 °C; specifically, the mass loss at high temperatures over 1000 °C reaches only 38%, compared to 78% for pure GO. It is clear from the current study that the oxygen functional groups on the surface of GO sheets have been effectively eliminated throughout the reduction process.

### 3.5. Raman Spectral Analysis

**Figure 5:**
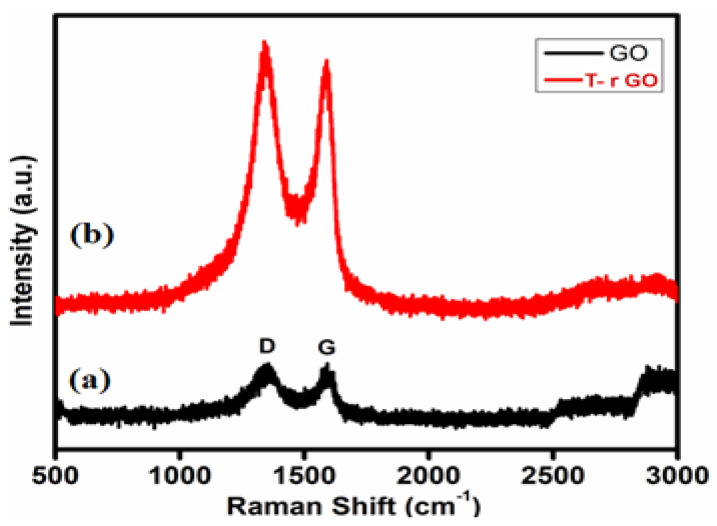
Raman spectra of (a) GO and (b) T-rGO.

Raman spectroscopy helps to identify graphene from graphite by looking at the variation in surface charge, structural faults, and layer counts. GO and T-rGO molecules were clearly differentiated by Raman spectroscopy, as highlighted in Figure 5. Both the G (E2g mode) and D (symmetric A1g mode) bands are observed at 1383 and 1576 cm^−1^, respectively, in the GO spectrum. The size of the *sp^2^* domain is found to decrease in-plane due to the intensification of the D-band achieved through the enhanced graphite oxidation.

Although the GO levels are decreased by thiourea, these bands remain visible in the T-rGO Raman spectrum. Furthermore, the I_D-band_/I_G-band_ ratio based on the Raman spectra is found to be 0.96 and 1.24 for GO and T-rGO, respectively. Due to the strong D and G bands at 1362 and 1563 cm^−1^, T-rGO exhibits a higher D/G intensity ratio (Figure 5) [46,47].

### 3.6. Antifungal Activity of Reduced Graphene Oxide Nanosheets

**Figure 6:**
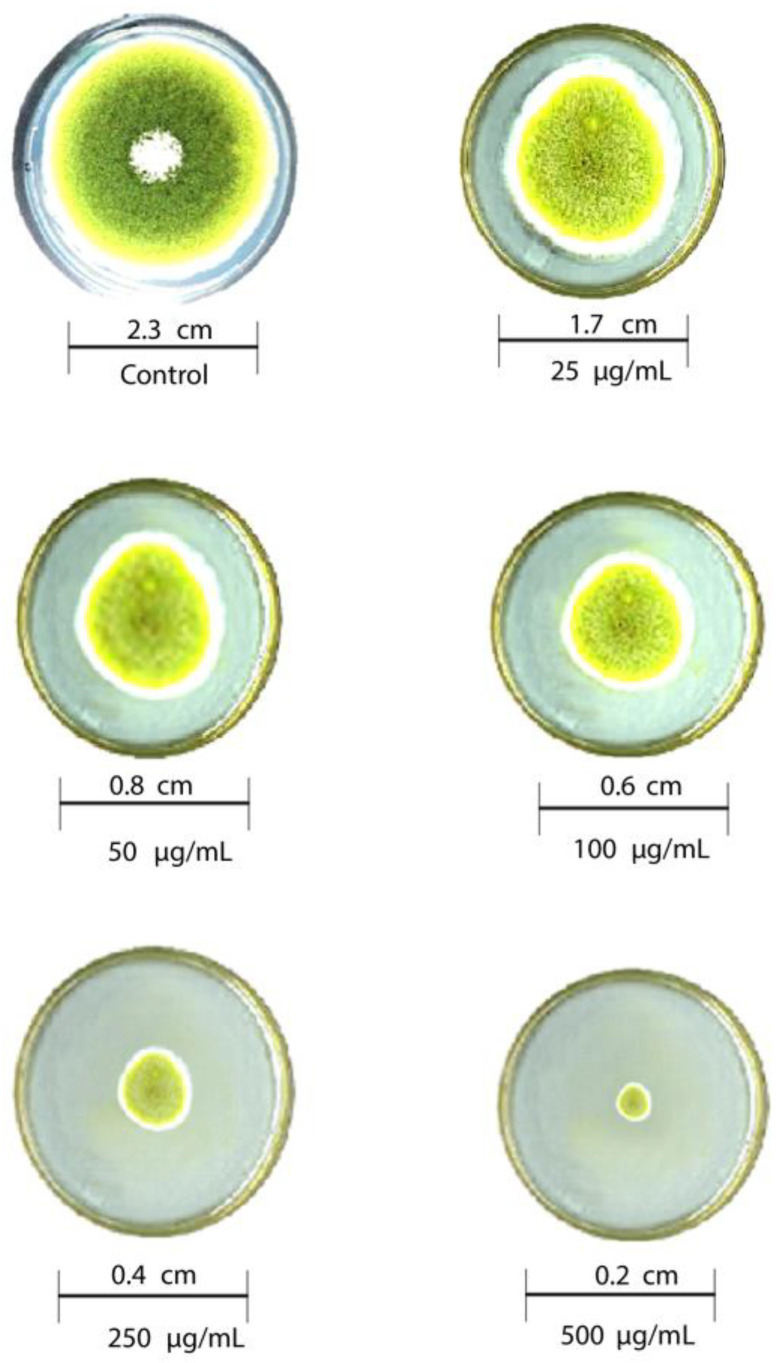
Antifungal activity of T-rGO nanosheets: mycelial growth of Aspergillus flavus on the PDA media containing different concentrations of reduced graphene oxide (25–500 µg/mL).

**Figure 7:**
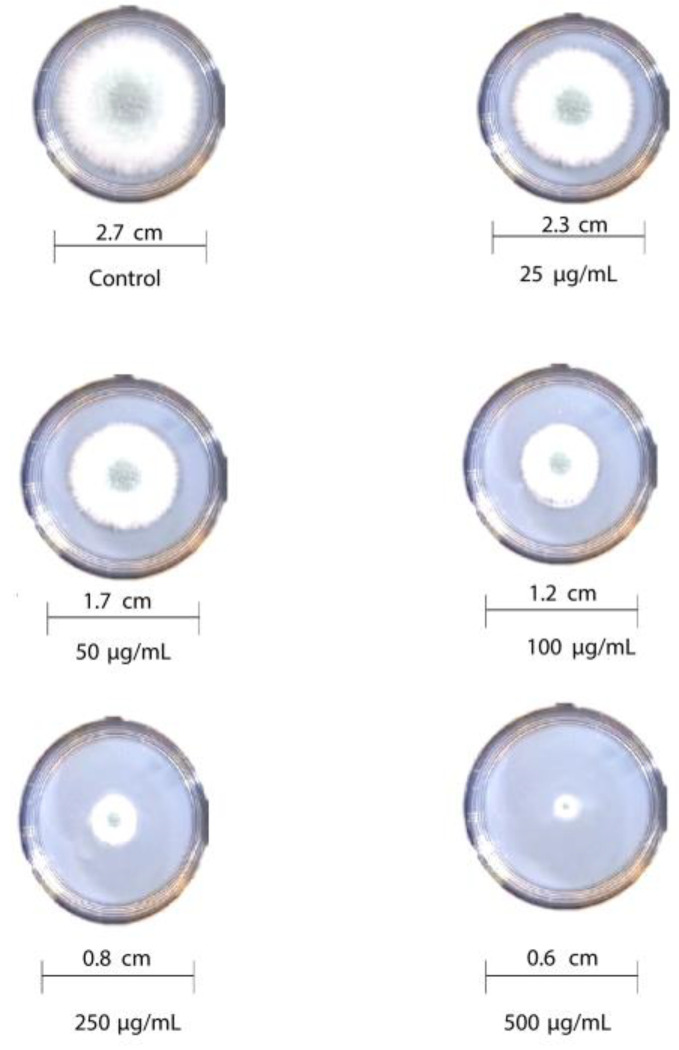
Antifungal activity of T-rGO nanosheets: mycelial growth of Aspergillus fumigatus on the PDA media containing different concentrations of reduced graphene oxide (25–500 µg/mL).

The antifungal activity of T-rGO against selected fungi is displayed in Figure 6 and Figure 7, respectively. It is clearly observed that T-rGO nanosheets at concentrations of 25–500 µg/mL completely prevent the growth of *A. flavus* and *A. fumigatus* mycelia. The direct interaction of T-rGO nanosheets with the cell walls of the fungi is most likely the cause for effectively inhibiting fungi cell synthesis, or most likely, the efficient inhibition of fungal cell wall formation is caused by T-rGO nanosheets direct interaction with the cell walls of the fungi. Chitin and other polysaccharides present on the cell walls of fungus may chemically react with the reactive oxygen-containing functionalities of a number of tiny reduced graphene oxide nanosheets [48]. Notably, it had previously been reported [49] that materials associated with graphene had antibacterial properties due to direct interaction with bacterial cells.

**Figure 8:**
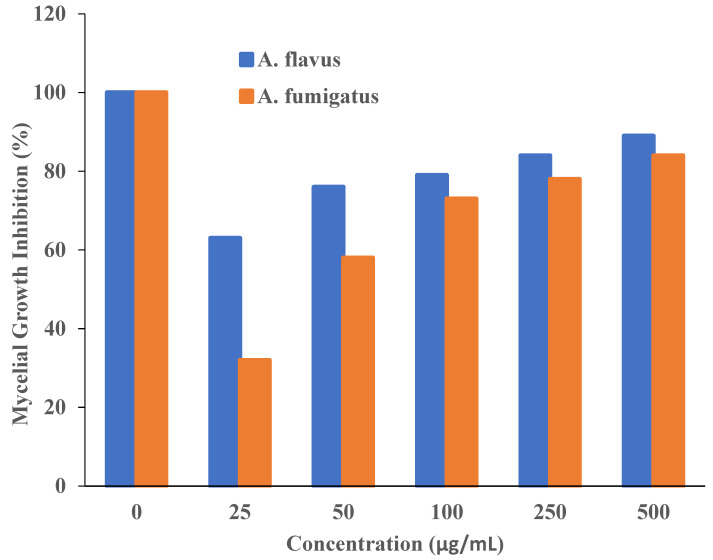
Plots of T-rGO concentration (µg/mL) vs. the activity that inhibits mycelial growth (percent).

Additionally, it was asserted that the antifungal properties of essential oils (for example, phenol) with oxygen species functional groups were caused by direct contact with the cell walls of fungi [50,51]. Since the amount of fungal growth inhibition was found to be dependent on the concentration, the half-maximal inhibitory concentration (IC50) values are determined by plotting the logarithm of the concentration of T-rGO (µg/mL) against the mycelia growth inhibitory activity (percent), as shown in Figure 8. It can be observed that T-rGO nanosheets’ IC_50_ values for *A. fumigatus* and *A. flavus* attain 100 to 500 µg/mL, respectively. This may be due to the fact that reduced graphene oxide nanosheets readily bind to fungi exterior cell walls via hydroxyl oxygen species of glycoproteins [52].

The obtained inhibitory concentrations are around two times lower than those of ZnO nanoparticles [53] and essential oils [50], which were both used as antifungal materials. This proves that the reduced graphene oxide nanosheets exhibit superior antifungal activity.

### 3.7. Effect of GO and T-rGO on A549 Human Lung Cancer Cells

In this study, the effectiveness of GO and T-rGO on the viability of human lung cancer cells (A549) was examined. In order to verify whether GO and T-rGO possess an inhibitory effect, the cells were treated with a broad concentration range of 1000−7.8 µg/mL for 24 h.

**Figure 9:**
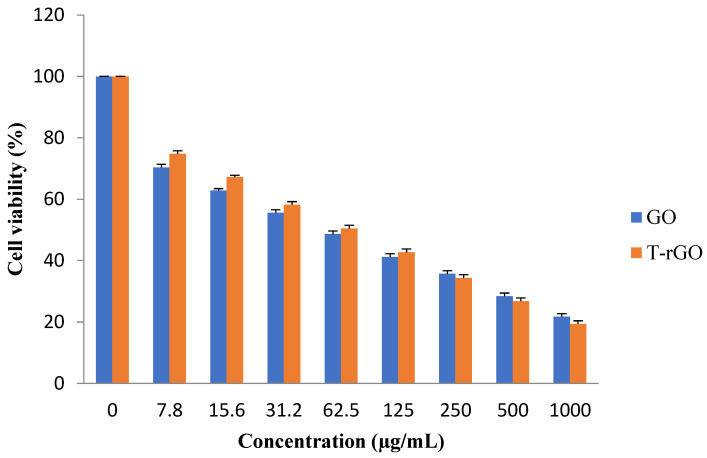
Effects of GO and T-rGO on cell viability of human lung cancer cells. The viability of human lung cancer cells (A549) was determined after 24 h exposure to different concentrations of GO and T-rGO using the WST-8 assay, (Mean ± SD).

The results reveal that the groups treated with GO, as shown in Figure 9, demonstrate higher cytotoxicity around 70.32% at a lower dose of 7.8 µg/mL, which increases up to 74.77% for the groups treated with T-rGO. With further increase in the concentration, the cytotoxicity decreases markedly and linearly for both GO and T-rGO, indicating a dose-dependent trend. Interestingly, irrespective of the concentration, T-rGO exhibits a greater cytotoxicity compared to GO up to the concentration of 125 µg/mL, and then the effect is reversed where GO cytotoxicity became more effective. At the highest dose of 1000 µg/mL after incubation for 24 h, the treatment with T-rGO reduces cell viability by up to 19.34%, while with GO, the cell mortality was enhanced, reaching 21.69%. According to Akhavan et al., both size and concentration of graphene-based materials were found to have harmful effects on human mesenchymal stem cells [54].

### 3.8. Graphene-Induced Apoptosis in A549 Human Lung Cancer Cell Line

**Figure 10:**
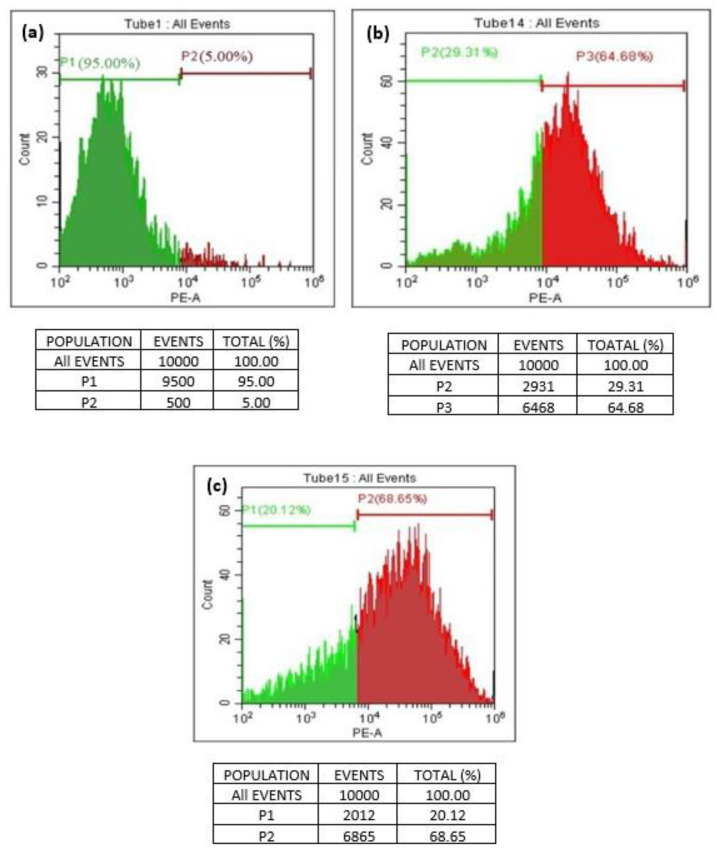
(**a**–**c**) Flowcytometric detection of apoptosis in phase of human lung cancer cells ((**a**): control; (**b**): GO-treated cells; (**c**): T-rGO-treated cells).

A quantitative in vitro apoptotic detection technique (flowcytometric or cell cycle analysis) was utilized to determine whether apoptosis plays a key factor in cell death induced by GO and T-rGO. The representative DNA histograms (Figure 10) were obtained following propidium iodide staining of permeabilized human lung cancer cells A549. The obtained results demonstrate that apoptosis is the primary mechanism responsible for the cell death produced by GO and T-rGO. This is in excellent agreement with the literature [55].

The percentage of live and dead cells versus a total of 100% cells manifested the cell cycle analysis. The dead cells and the living cells have been distinguished by a line on a scale of 10^2^ to 10^6^. The cells between 10^2^ and 10^4^ are found alive, whereas the cells exceeding 10^5^ are found dead.

### 3.9. DNA Laddering

**Figure 11:**
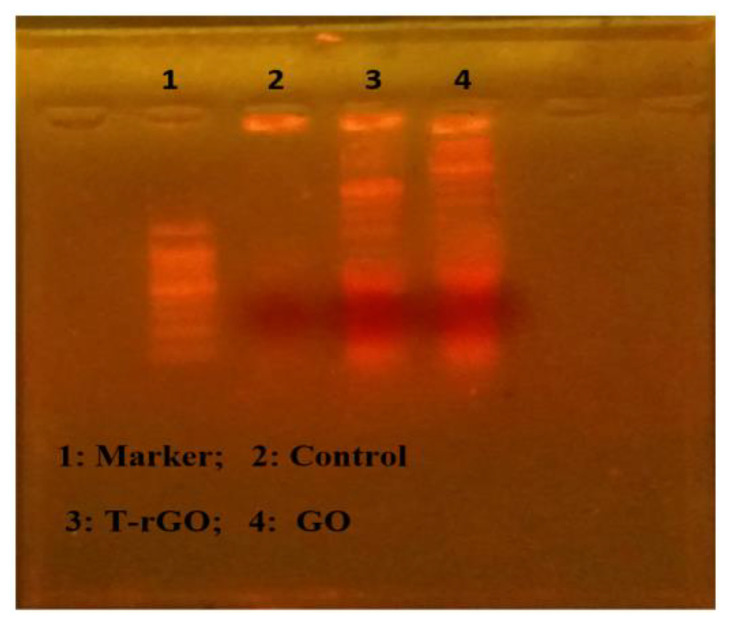
DNA fragmentation. Cells were incubated with GO (62.5 µg/mL) and T-rGO (62.5 µg/mL) for 24 h; after incubation, DNA was extracted from cells and resolved on agarose gel electrophoresis.

The apoptotic processes occurring before chromosomal DNA fragmentation, a defining characteristic of apoptosis, were conducted to evaluate how much apoptosis GO and T-rGO cause in human lung cancer cells A549. DNA fragmentation was then used to examine the effects of GO and T-rGO treatments on the cells. The results of the DNA fragmentation analysis were found to be essentially similar for all samples, as illustrated in Figure 11. In the literature, it was reported that by activating caspase 8 and caspase 3, the in vivo study found that daunorubicin-loaded graphene–gold nanocomposites caused apoptosis and inhibited the growth of multidrug-resistant leukemia cells [37]. The graphene-based materials caused DNA fragmentation in cancer cells [54].

### 3.10. Cell Morphology

Additionally, the effect of cytotoxicity is investigated using an inverted microscope, by examining the morphology of human lung cancer cell A549 before and after GO and T-rGO treatment. Indeed, it was reported in the literature that significant time-dependent morphological alterations were caused by GO and T-rGO, such as cell shape loss, rupture of the monolayer, and decreased cell adhesion, pointing to compromised cell viability.

**Figure 12:**
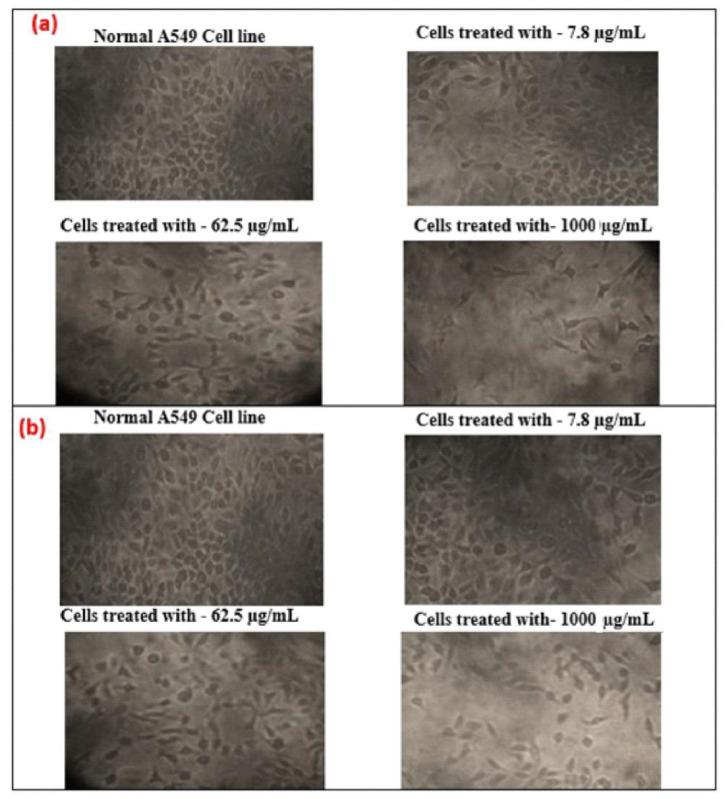
(**a**) Anticancer effect of GO on A549 human lung cancer cells; (**b**) anticancer effect of T-rGO on A549 human lung cancer cells.

The effect on the morphology of cells as a function of dose (1000, 62.5, and 7.8 µg/mL) on human lung cancer cell monolayers cultured with and without GO and T-rGO (IC_50_ concentration) over 24 h is shown in Figure 12. A definite distinction can be observed between the control cells compared to GO and T-rGO treated cells. The morphology of treated cells is altered; they become very thin and feeble, and develop elongated tips. The untreated cells are found to remain healthy. These findings are in good agreement with the effect of resveratrol on ovarian cancer cells as reported in the literature [56].

## 4. Conclusions

Presently, diverse fields of applications stand to benefit from the fascinating tunable properties of graphene and its derivatives. In the current study, graphene oxide was reduced by the reducing and stabilizing agent thiourea to produce graphene via a chemical process. The as-fabricated GO and T-rGO were identified using X-ray diffraction, TGA, FTIR, SEM, and Raman spectroscopy, and indicated the growth of graphene when GO’s oxygen-containing functional groups were removed from its surface. The platelet shape and the steady transparent character of graphene were confirmed by SEM observations, while thermogravimetric analysis manifested its improved thermal stability. This demonstrated that this method offers a simple cost-effective route to upscale the fabrication of graphene.

Additionally, reduced graphene oxide’s efficacy as an antifungal was tested against two plant-pathogenic fungus, *A. flavus* and *A. fumigatus*. Furthermore, cell viability and cell cycle analysis, as well as DNA laddering, indicated that GO was more cytotoxic to the cells than T-rGO. According to the present research, reduced graphene oxide proved to be a promising nanomaterial for combating fungus.

## Data Availability

Not applicable.
